# Changes in Eating Competence and Its Association with Dietary and Clinical Outcomes in Brazilian Older Adults with Type 2 Diabetes or Prediabetes Attending a Public Health Service

**DOI:** 10.3390/nu18213605

**Published:** 2026-10-30

**Authors:** Rafaella Dusi, Raíza Rana de Souza Lima Trombini, Silvana Schwerz Funghetto, Marina Morato Stival, Eduardo Yoshio Nakano, Cláudia Chaves, Renata Puppin Zandonadi

**Affiliations:** 1 Department of Nutrition, Faculty of Health Sciences, University of Brasília, Campus Universitario Darcy Ribeiro, Brasilia 70910-900, Brazil; 2 Graduate Program in Health Sciences and Technologies, University of Brasília, Campus Universitario Ceilândia, Brasilia 72220-275, Brazil; 3 Department of Statistics, University of Brasília, Campus Universitario Darcy Ribeiro, Brasilia 70910-900, Brazil; 4 School of Health (ESSV), Polytechnic University of Viseu, 3504-510 Viseu, Portugal

**Keywords:** nutrition, type 2 diabetes mellitus, primary health care, older adults, eating competence, food and nutrition education

## Abstract

**Background/Objectives:** This prospective, longitudinal, quasi-experimental study evaluated changes in eating competence (EatC) and its association with nutritional risk, dietary markers, and clinical outcomes, including body mass index (BMI), body fat percentage, and glycated hemoglobin (HbA1c), in Brazilian older adults with type 2 diabetes (T2DM) or prediabetes. **Methods:** Thirty-three older adults (87.9% female, predominantly inactive, regarding occupational status, and with low educational level) participated in a 12-month food and nutrition education (FNE) program at a public Primary Health Care unit in Ceilândia, Distrito Federal, Brazil. EatC was assessed using the validated ecSI2.0™BR; nutritional risk was measured by the Determine Your Nutritional Health (DNH) Checklist; and dietary intake was characterized using VIGITEL markers. Body composition was measured by bioelectrical impedance and HbA1c using standardized methods. **Results:** At baseline, regarding occupational status, active participants had significantly higher total EatC scores than inactive participants (*p* = 0.021). After 12 months, body fat percentage decreased significantly (*p* = 0.011), whereas BMI and HbA1c remained stable. Total EatC scores and its domains did not change significantly and most participants were already competent eaters at baseline (84.8%). However, variation in specific EatC domains was associated with important outcomes. Variation in food acceptance was negatively correlated with DNH variation (r = −0.417, *p* = 0.016), indicating an association between increased food acceptance and reduced nutritional risk. Variation in internal regulation was positively correlated with DNH variation (r = 0.492, *p* = 0.004). Variation in contextual skills was negatively associated with variation in body fat percentage (r = −0.412, *p* = 0.019). Additionally, variation in soda or artificial juice consumption was positively associated with variation in nutritional risk (r = 0.503, *p* = 0.003). **Conclusions:** Improvements in contextual skills and food acceptance were associated with better clinical and nutritional outcomes in older adults with T2DM or prediabetes. Public health interventions should prioritize meal planning, food preparation, and contextual skills as factors potentially associated with nutritional risk.

## 1. Introduction

Diabetes Mellitus (DM) is a disease characterized by persistently elevated blood glucose levels, insufficient insulin production, and/or impaired insulin utilization. Type 2 diabetes mellitus (T2DM) is the most common form of DM, accounting for more than 90% of diagnosed cases. T2DM represents a major global public health concern, with a growing number of individuals affected: in 2024, an estimated 589 million adults were affected, with projections reaching approximately 853 million by 2050 [1,2]. T2DM is typically diagnosed after the third decade of life, and the condition may be preventable or delayed. Its often asymptomatic presentation frequently contributes to delayed diagnosis, and it is estimated that between 30% and 50% of individuals with T2DM in Brazil are unaware of their condition, increasing the risk of complications such as cardiovascular and ophthalmologic diseases [1,2,3,4].

In Brazil, the self-reported prevalence of T2DM among adults reached 12.9% in 2024 [5,6,7], with an estimated 16.6 million adults affected and projections of 24 million by 2050 [1,2], driven by an aging population and factors such as obesity, hypertension, and low educational levels. The impact is most severe among older adults, who have higher rates of hospitalization (ages 60–69) and mortality (over 70 years) [5], as well as complications such as lower limb injuries, ophthalmic and renal diseases, strokes, depression, and mood disorders [8,9], sarcopenia, and difficulties with dietary control and medication use, with a high risk of severe hypoglycemia. In addition, there is the emotional distress associated with T2DM (diabetes distress), often related to low adherence to dietary self-care [8,10] exacerbated by conventional approaches that focus on prescriptive dietary restrictions, which generate negative feelings and compromise patient engagement [11,12] reinforcing the need for empathy-based, non-prescriptive food and nutrition education (FNE) initiatives focused on empowerment and quality of life [8].

Prediabetes is characterized by blood glucose levels above normal but below the diagnostic criteria for T2DM [13]. Available data on the prevalence of prediabetes shows an estimate that 635 million adults aged 20 to 79 around the world had impaired glucose tolerance and that 488 million had impaired fasting glucose. In Brazil, the prevalence of impaired glucose tolerance is 11.3%, and impaired fasting glucose is 9.7% [1]. Individuals with prediabetes have an increased risk of developing T2DM, and the risk of progression increases when glycated hemoglobin (HbA1c) levels approach the diagnostic cut-off for T2DM [13]. Health education and lifestyle interventions, including changes in eating habits, are recommended for individuals with prediabetes and can help prevent or delay the development of T2DM [14,15]. Furthermore, strategies that promote healthy eating are recommended for both individuals with prediabetes and those with a confirmed diagnosis of T2DM [16].

Given these concerns, dietary management strategies are indispensable for glycemic control and reducing prediabetes or T2DM-associated complications. Nutritional recommendations for patients with prediabetes or T2DM aim to manage blood glucose, body weight, and blood pressure, promoting eating habits that reduce the risk of complications from decompensation [17]. Adequate nutrition is essential for prevention and control of the disease; however, to achieve these objectives, nutritional guidance must consider each patient’s dietary preferences and cultural habits. This approach can improve adherence to dietary treatment while promoting self-care and individual autonomy [3,17,18,19,20]. In this sense, the Eating Competence (EatC) model proposes a biopsychosocial approach focused on four domains: eating attitudes (a positive and flexible interest in food), food acceptance (the ability to accept and experiment with a variety of foods), internal regulation (awareness of internal hunger, appetite, and satiety signals), and contextual skills (resources for planning and organizing meals) [21,22]. This approach aims to promote autonomy and flexibility in individual choices, allowing eating to be experienced with pleasure, confidence, and self-regulation [21]. This perspective aligns with FNE strategies that encourage active participation, value self-care, and respect food culture [18,19].

Adopting continuous educational approaches based on autonomy within Primary Health Care is essential [14]. Food and nutrition guidance and longitudinal care are fundamental strategies to prevent negative clinical outcomes and to reduce the costs that decompensated disease imposes on the country [14]. Because of this clinical and financial impact, guidelines recommend that health education be a continuous and periodic process within the public health system. However, it is essential to evaluate whether, in practice, these nutritional and behavioral guidelines have a positive effect after the 12-month intervention when incorporated into the daily routine of the public health system [18]. A systematic review and meta-analysis showed that FNE interventions lasting at least three months improve glycated hemoglobin control in patients with T2DM, and that approaches that include psychosocial aspects are more effective than those focused solely on nutrients [23].

In Brazil, a study of adults investigated the association between EatC, food consumption, and health outcomes [24]. Another Brazilian study examined the relationship between cooking and food skills and EatC in adults [25]. Similarly, a study of students from public and private universities across the five Brazilian regions evaluated EatC and its association with health indicators [26]. In addition, a Brazilian study examined changes in EatC among adults by comparing periods before and during the COVID-19 pandemic [27]. To date, no study has evaluated EatC or its association with nutritional risk, dietary markers, or clinical outcomes in older adults with T2DM or prediabetes.

Considering the studies cited above, in this sense, we hypothesized that older adults with type 2 diabetes or prediabetes undergoing longitudinal follow-up—including food and nutrition education—within Primary Health Care will show improved EatC over 12 months, and that this improvement will be associated with better dietary markers, nutritional risk, and clinical outcomes, specifically lower BMI, lower body fat percentage, and lower glycated hemoglobin. However, a gap remains in Brazil regarding the impact of Primary Health Care monitoring on glycated hemoglobin control and EatC among older adults with T2DM or prediabetes. This motivates the present study, which aims to evaluate the temporal evolution of EatC and its association with nutritional risk, dietary markers, and clinical outcomes (BMI, body fat percentage, and glycated hemoglobin) in older adults with T2DM or prediabetes treated at a public health service. Given the potential of FNE, as outlined by the EatC model for T2DM and prediabetes prevention and management, and considering the rising incidence of the disease and population aging in Brazil, there is a notable lack of studies investigating this approach in the Brazilian context.

## 2. Materials and Methods

### 2.1. Study Design

This prospective, longitudinal, quasi-experimental intervention study employed a single-group pre-post design to evaluate changes in EatC, dietary markers, nutritional risk, and clinical outcomes at baseline and after twelve months of a structured food and nutrition education (FNE) program delivered in a Primary Health Care unit linked to the Brazilian Unified Health System.

### 2.2. Settings and Intervention

This study was conducted between September 2024 and September 2025 at a public Primary Health Care unit in Ceilândia, Distrito Federal, Brazil.

A standardized food and nutrition education program was implemented over 12 months starting with a baseline meeting to provide a summary of the dietary guidelines contained in the official protocols: Protocol for the Use of the Dietary Guidelines for the Brazilian Population: Dietary Guidance for Adults with Obesity, Arterial Hypertension, and Diabetes Mellitus (*Protocolo de Uso do Guia Alimentar para a População Brasileira: Orientação Alimentar de Pessoas Adultas com Obesidade, Hipertensão Arterial e Diabetes Mellitus*), Protocol for the Use of the Dietary Guidelines for the Brazilian Population in the Dietary Guidance for the Older adults (*Protocolo de Uso do Guia Alimentar para a População Brasileira na Orientação Alimentar da Pessoa Idosa*), and the manual Cardioprotective Diet: Guidance Manual for Health Professionals in Primary Care (*Alimentação Cardioprotetora: Manual de Orientações para os Profissionais de Saúde da Atenção Básica*), in addition to the Dietary Guidelines for the Brazilian Population (*Guia Alimentar para a População Brasileira*) [14,28,29,30]. At this baseline meeting, participants received a kit of standardized measuring cups and spoons to support adherence to recommendations in daily routines and to allow adequate food portioning. Educational and guiding materials focusing on eating habits, food choices, and meal preparation were also provided from the very first encounter for participants to consult at home.

Following this initial stage, longitudinal follow-up included three additional in-person meetings, spaced 90 days apart, concluding with the final assessment twelve months after the baseline intervention. Food and nutrition education interventions were conducted at the Primary Health Care unit through facilitated group discussions designed to encourage the exchange of experiences, active participation, and the clarification of participants’ questions regarding diet, food, and health. The sessions were conducted by a registered dietitian, with support from a nurse and a team of undergraduate nutrition students.

The follow-up meetings focused on explaining the use of household measures to assemble healthy meals. The gram equivalents for portions of carbohydrates, such as root vegetables and corn couscous, and proteins, such as ground beef, eggs, and legumes (beans) were detailed. The older adults learned to use the kit to compose balanced meals, independently controlling the volume of each meal. Examples of breakfast, lunch, snacks, and dinner were discussed using the household measures and gram equivalents provided in the supporting material.

The meetings also addressed the distinction between food processing levels, critical label reading, including terms such as “diet,” “light,” and “fit” on food packages, and the importance of habits such as mindful eating in quiet environments. Participants were encouraged to eat slowly, avoid screens during meals, and eat with others when possible. The recommendations also included planning purchases at grocery stores and farmers’ markets, choosing seasonal foods, preparing simple recipes, and freezing homemade meals to make daily eating routine easier. Special emphasis was placed on demystifying the consumption of fruits and carbohydrates by discussing differences between whole fruits and juices, portion sizes, and food combinations as strategies to reduce glycemic load. Researchers instructed participants never to consume these foods in isolation, recommending they always be combined with sources of fiber, protein, or high-quality fats to prevent glucose spikes and prolong satiety. Throughout the entire process, actions aimed to optimize self-care routines and food preparation, promoting autonomy and flexibility in individual choices in line with the EatC model [21].

### 2.3. Study Size and Participants

The study recruited all eligible individuals from the local institution rather than relying on a convenience sample. Since the aim of this study was to evaluate the temporal evolution of EatC and its association with nutritional risk, dietary markers, and clinical outcomes in older adults with T2DM or prediabetes treated at a public health service, and this service does not attend healthy older adults, a single group (without a control group) of eligible participants was assessed at baseline and after the 12-month intervention. Eligibility criteria required that participants be aged 60–79 years, have a diagnosis of type 2 diabetes or prediabetes, be literate, own a mobile phone with access to WhatsApp, and demonstrate independence and autonomy in daily life activities, even when adaptation or supervision from third parties was required. Exclusion criteria comprised a diagnosis of type 1 diabetes, insulin use, chronic complications of diabetes, including renal failure, retinopathy, neuropathy, limb amputation, or diabetic foot, mobility limitations, moderate or severe cognitive impairment, and stage 3 hypertension. Initially, the researchers accessed records of potential participants aged 60 years or older diagnosed with T2DM or prediabetes at the Primary Health Care unit, yielding a total reference population of 187 older adults.

Active telephone screening was systematically conducted for all 187 identified individuals. Each candidate was contacted up to three times at different times. During these calls, specific exclusion criteria for this stage of the research were applied: individuals were excluded if they could not be reached after three separate telephone attempts; they expressed a lack of interest in participating after a detailed explanation of the study’s objectives; or they reported being on insulin therapy at the time of the initial clinical screening.

**Figure 1:**
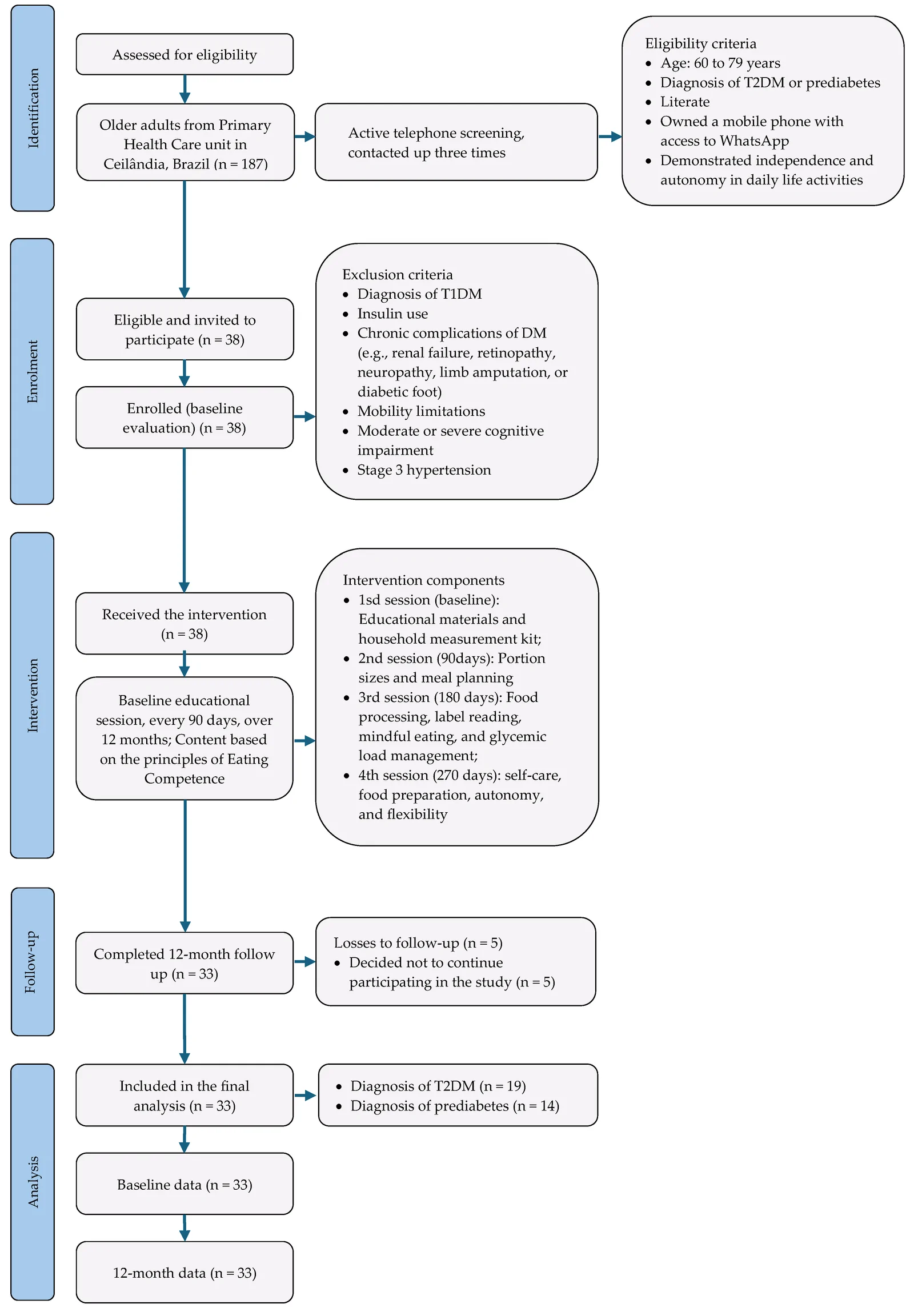
Flowchart of participant recruitment and follow-up throughout the 12-month study.

After meeting the eligibility criteria, eligible participants signed the Free and Informed Consent Form (FICF) before enrollment in the study. In total, 38 participants were enrolled before the intervention began. During the study, 5 of the 38 enrolled participants did not complete either assessment period and were subsequently excluded from the longitudinal dataset. The final analytic sample therefore consisted of 33 participants who completed the full set of assessment instruments at both baseline and the 12-month follow-up. Of these, 19 (57.6%) had a diagnosis of T2DM and 14 (42.4%) had prediabetes. Figure 1 summarizes participant recruitment and follow-up throughout the 12-month quasi-experimental study.

### 2.4. Variables

Data collection included behavioral, nutritional, and clinical variables. The primary outcome was EatC, and the secondary outcomes were HbA1c, BMI, body fat, DNH, and dietary markers. Participant progress was monitored at 0 and 12 months using dietary intake markers and clinical outcomes, including glycated hemoglobin levels, body mass index (BMI), and body fat percentage obtained via bioimpedance. EatC was assessed using the *ecSI2.0™BR* questionnaire [22]. The ecSI2.0™BR consists of 16 items assessed using a five-point Likert scale ranging from always to never (always, often, sometimes, rarely, and never, scored as 3, 2, 1, 0, and 0 points, respectively), with a total score ranging from 0 to 48. Scores of 32 or higher classify individuals as competent eaters, and higher scores indicate greater EatC [21,22,31,32].

Nutritional risk was assessed using the Determine Your Nutritional Health (DNH) Checklist, a 10-item instrument that measures poor nutrition warning signs. Each “yes” answer is weighted from 1 to 4 points (with a “no” answer worth 0 points), resulting in a total score ranging from 0 to 21. The exact checklist items and their respective scores for a “yes” response are: “I have an illness or condition that made me change the kind and /or amount of food I eat” (2 points); “I eat fewer than two meals per day” (3 points); “I eat few fruits or vegetables, or milk products” (2 points); “I have three or more drinks of beer, liquor or wine almost every day” (2 points); “I have tooth or mouth problems that make it hard for me to eat” (2 points); “I don’t always have enough money to buy the food I need” (4 points); “I eat alone most of the time” (1 point); “I take three or more different prescribed or over-the-counter drugs a day” (1 point); “Without wanting to, I have lost or gained 10 pounds in the last six months” (2 points); and “I am not always physically able to shop, cook and/or feed myself” (2 points). Based on the total score, older adults were classified into three nutritional risk levels: 0 to 2 points indicate good nutritional health or low risk, 3 to 5 points indicate moderate nutritional risk, and 6 or more points indicate high nutritional risk [33,34,35,36,37]. Food consumption markers were extracted from questions in the VIGITEL, a Brazilian telephone survey for the surveillance of risk and protective factors for chronic diseases. This instrument defines regular dietary intake as occurring at least five days per week. The evaluated items were divided into healthy dietary markers (beans, vegetables, salad or raw vegetables, natural fruit juice, and fruits) and unhealthy dietary markers (soda or artificial juices) [6,7]. To evaluate nutritional status, the body mass index (BMI) [38] was calculated from weight, measured using the built-in scale of the bioelectrical impedance (InBody 270, InBody Co., Ltd., Seoul, Republic of Korea) [39], and height, measured using a stadiometer and classified according to BMI cut-off points for older adults: underweight (<22 kg/m^2^), normal weight (22 to 27 kg/m^2^), or overweight (>27 kg/m^2^) [10].

Body fat percentage was measured via bioelectrical impedance (InBody 270, InBody Co., Ltd., Seoul, Republic of Korea) [39], a non-invasive body composition method applicable to older adults [40]. The two bioimpedance assessments occurred early in the morning. Participants were instructed to fast for 8 to 12 h, not drink water for 3 h before the assessment, wear light clothing, and remove any metal accessories. Glycated hemoglobin (HbA1c) levels were measured in whole blood collected with ethylenediaminetetraacetic acid (EDTA) as an anticoagulant, using high-performance liquid chromatography (HPLC), with a method certified by the National Glycohemoglobin Standardization Program (NGSP) [13,41]. The measurements were also used to confirm whether participants had T2DM or prediabetes. For the clinical profile, time since diagnosis for T2DM among affected participants was 5.39 years (SD = 4.30 years), while time since diagnosis for prediabetes was 3.71 years (SD = 2.83 years).

### 2.5. Statistical Methods

For data analysis, cross-sectional analyses were initially performed at 0 months, using unpaired Student’s *t*-test to compare EatC scores with sociodemographic variables, and Pearson and Spearman correlations to cross-reference data from the *ecSI2.0™BR*, *DNH*, and *VIGITEL*. The temporal evolution from 0 to 12 months was evaluated using the paired Student’s *t*-test, McNemar’s test, and the Wilcoxon test, depending on the distribution of the variables. Finally, delta variations were analyzed using Pearson or Spearman correlations to determine whether increases in EatC were associated with improvements in nutritional risk or health outcomes among the older adults monitored. Statistical analyses were performed using the IBM Statistical Package for Social Sciences software version 25.0 (SPSS Inc., Chicago, IL, USA) and statistical significance was set at *p* < 0.05.

### 2.6. Ethical Aspects

This study was conducted in accordance with the Declaration of Helsinki and approved by the Research Ethics Committees of the Faculty of Ceilândia of the University of Brasília—CEP/FCE (5.142.057 and CAAE: 51915021.9.0000.8093) and the Foundation for Teaching and Research in Health Sciences—CEP/FEPECS (5.237.938 and CAAE: 51915021.9.0000.8093) on 2 December 2021.

## 3. Results

### 3.1. Participants’ Characteristics

Of the 38 potential participants, 33 older adults (19 with T2DM and 14 with prediabetes) fully completed the assessment instruments at both follow-up periods (0 and 12 months) and formed the final sample, with 29 females (87.9%) and 4 males (12.1%) (Figure 1). Most participants were women, retired, and had low educational attainment (Table 1). Baseline data showed associations between EatC scores and participants’ sociodemographic characteristics. Individuals with 9 or more years of education (*n* = 16) had higher food acceptance scores (*p* = 0.002), whereas those with lower educational attainment had higher contextual skills scores (*p* = 0.032) (Table 1). EatC scores did not differ significantly across age groups at baseline (Table 1).

Regarding professional occupation, older adults classified as active (*n* = 8) presented higher mean scores than inactive or retired individuals (*n* = 24) in relation to eating attitudes (*p* = 0.020), internal regulation (*p* = 0.024), and the total EatC score (active: mean of 42.13 and SD = 4.49 and inactive: mean of 36.42 and SD = 6.09; *p* = 0.021). No statistically significant differences in nutritional risk, as assessed by the DNH score, were detected by age group, education, and occupation at baseline.

### 3.2. Changes After Intervention

Over the 12-month intervention, body fat percentage significantly decreased (*p* = 0.011), whereas BMI and glycated hemoglobin remained stable (Table 2). EatC domains showed distinct patterns of change after the 12-month intervention (Table 2).

The variations in mean scores and frequency distributions of EatC, nutritional risk of the older adult, and food consumption markers did not reach statistical significance after the 12-month intervention (*p* > 0.05). Most participants were already classified as competent eaters (score ≥ 32) at baseline, with mean total EatC scores above the established cut-off in both assessments. Changes in the number of competent eaters, in mean DNH scores, and in nutritional risk classification did not differ statistically (Table 2). Additionally, consumption frequencies for healthy dietary markers (beans, vegetables, salad or raw vegetables, natural fruit juice, and fruits) and unhealthy dietary markers (soda or artificial juice), as assessed by VIGITEL items, did not change significantly after the 12-month intervention (Table 2).

### 3.3. Associations Between Eating Competence, Nutritional Risk, and Dietary Markers Consumption

Results showed no statistically significant association between the total ecSI2.0™BR and domain scores and the DNH risk score at either the first (r = −0.274; *p* = 0.123) or the last collection (r = −0.266; *p* = 0.135) (Table 3).

Concerning the healthy eating markers evaluated by VIGITEL, the data described in Table 4 demonstrate that the correlation analysis at the first collection highlighted a positive association between total EatC and the frequency of consumption of vegetables (Spearman correlation of 0.459; *p* = 0.007) and salad or raw vegetables (Spearman correlation of 0.420; *p* = 0.015). At the last collection, a negative association was identified between the salad or raw vegetable consumption marker and the DNH score; e.g., salad or raw vegetable consumption was negatively associated with nutritional risk, with a Spearman correlation of −0.484 and *p* = 0.004. No other dietary markers were significantly associated with EatC or DNH.

### 3.4. Associations Between Changes After the 12-Month Intervention

The relationship between variation in total EatC and variation in nutritional risk was not significant. Food acceptance presented a negative correlation with DNH variation, with a Pearson correlation coefficient of −0.417 and *p* = 0.016, indicating that increased food acceptance was associated with reduced nutritional risk (Table 5). In contrast, variation in internal regulation showed a positive correlation with DNH (Pearson correlation coefficient of 0.492 and *p* = 0.004) (Table 5).

Variation in soda or artificial juice intake frequency correlated positively and significantly with variation in nutritional risk (Spearman’s correlation coefficient = 0.503; *p* = 0.003), indicating that participants who increased soda consumption also increased nutritional risk (DNH). The remaining correlations between consumption changes and modifications in total ecSI2.0™BR and DNH scores did not reach statistical significance (Table 6).

### 3.5. Associations with Clinical Outcomes and Their Variations After the 12-Month Intervention

The analysis of EatC scores and clinical outcomes showed that internal regulation presented a positive correlation with BMI at the first collection, with a Pearson correlation coefficient of 0.424 and *p* = 0.014. No other significant associations were identified (Table 7).

Table 8 shows that the variation in contextual skills scores is negatively and statistically significantly correlated with the variation in body fat percentage. This cross-reference showed a Pearson correlation coefficient of −0.412 and *p* = 0.019, indicating that individuals who increased their contextual skills also showed a decrease in their body fat percentage. No significant associations were observed between changes in EatC and changes in HbA1c or BMI.

## 4. Discussion

To our knowledge, this is the first Brazilian longitudinal study to evaluate EatC among older adults with T2DM or prediabetes participating in a structured FNE program in public Primary Health Care. Using the validated instruments and a prospective 12-month follow-up, this study investigated longitudinal changes in EatC and their associations with dietary markers, nutritional risk, and clinical outcomes. The main findings showed that changes in specific EatC domains, rather than in the total EatC score, were associated with favorable outcomes. Greater improvements in contextual skills were associated with reductions in body fat percentage, and greater improvements in the food acceptance domain were associated with lower nutritional risk. Most participants were already classified as competent eaters at baseline, with mean EatC scores above the established cut-off (≥32) and nearly 85% of the sample meeting the criterion for competent eaters before the intervention (Table 2). Consequently, the limited room for improvement in the total score may have reduced the instrument’s ability to detect additional gains over time, despite favorable changes in body fat percentage and specific EatC domains.

### 4.1. Participants Characteristics

Baseline data showed that older adults with 9 or more years of education had higher food acceptance scores, but in the contextual skills domain, the lower-education group had a higher mean score. The association of educational level with the scores reflects the importance of health education. As shown by a Brazilian study that aimed to estimate the prevalence of complications linked to T2DM, and another that investigated inequalities in care for people with diabetes, lower educational level hinders understanding of the disease and engagement in self-care, limiting positive eating attitudes and treatment adherence [42,43].

Regarding professional occupation, the subjects classified as active showed higher mean scores for eating attitudes, internal regulation and the total EatC score. This aligns with an integrative literature review on the contribution of the Brazilian Unified Health System to the control of T2DM, which suggests that inactivity (common in retirement) can worsen the vulnerability of older adults, and shows that inactive diabetic patients report greater suffering that negatively impacts eating [44]. This emotional suffering can decrease motivation for the adoption of healthy habits and can favor “emotional eating”, as exposed by another Brazilian study on emotional suffering and adherence to self-care activities in older adults with T2DM [12]. In contrast, evidence indicates that a higher EatC score is frequently associated with a lower occurrence of emotional eating [45].

In the context of our study, it is essential to reinforce that participants already started the intervention with an elevated EatC. This may have acted as a protective factor, helping these older adults cope better with the emotional distress and disordered eating behaviors commonly associated with T2DM and inactivity. Evidence supports this protective role, indicating that competent eaters show lower emotional and uncontrolled eating, along with lower perceived stress [24,31]. In addition, studies in Brazilian adults show that higher EatC is associated with fewer eating disorder-related behaviors and greater confidence in making food choices [32]. Furthermore, recent evidence shows that lower EatC is associated with a higher severity of depressive symptoms, reinforcing that our participants’ high baseline EatC may have shielded them from these psychological burdens [46]. This suggests that maintaining this high level of EatC may help protect participants from emotional eating and the psychosocial distress associated with T2DM.

### 4.2. Changes After Intervention

Based on the results, the mean BMI and glycated hemoglobin (HbA1c) levels did not differ significantly after the 12-month intervention. Since the participants already had good baseline glycemic control, as indicated by a mean HbA1c of approximately 6% [14,47], there was little room for further meaningful reductions. According to a Brazilian clinical protocol and therapeutic guideline for T2DM and the American Diabetes Association Standards of Care, the established HbA1c target is <7.5% for healthy older adults and <8.0% for compromised older adults [14,47], confirming that our sample had good baseline glycemic control. Consistent with these findings, a systematic review with meta-analysis evaluating the impact of dietary education interventions on individuals with T2DM highlighted the difficulty of significantly improving metabolic profiles in primary care [23]. This good baseline glycemic control starting point, combined with a 12-month follow-up period, may have naturally limited the detection of further metabolic improvements.

Despite stable BMI and HbA1c, participants’ mean body fat percentage decreased significantly from baseline to the end of the study. This finding has important clinical implications, since weight reduction or BMI alone may not accurately reflect improvements in this population’s health. In our study, this may reflect reduced adiposity without necessarily reducing overall weight, which may be relevant to metabolic health, because excessive body fat is associated with insulin resistance [48]. As shown in a Brazilian study on the prevalence and incidence of T2DM in older adults, aging is naturally associated with fat accumulation in muscle tissue (replacing healthy muscle mass), which is directly linked to increased insulin resistance and glucose intolerance [49]. Furthermore, the literature shows that excess adipose tissue can contribute to increased insulin resistance in T2DM and prediabetes, as demonstrated in a Brazilian study that analyzed data from the 2019 National Health Survey [42], and corroborated by another study, also conducted in Brazil, that investigated the prevalence of prediabetes and intermediate hyperglycemia in adults [50].

The behavioral aspect of this improvement in body composition may relate to the development of contextual skills in older adults, since higher scores in this domain were associated with lower body fat percentage. This finding is aligned with a review of observational studies exploring the relationship between health and non-weight-centric eating behaviors, including EatC, which, among the findings, showed association with lower adiposity measures [45].

### 4.3. Associations Between Eating Competence, Nutritional Risk, and Dietary Markers Consumption

Results did not show a statistically significant association between the total ecSI2.0™BR scores and the DNH risk score. For the healthy eating markers evaluated by VIGITEL, the data demonstrate a positive association between total EatC and the frequency of consumption of vegetables and salads or raw vegetables. In the last collection, a negative association was identified between the salad or raw vegetable consumption marker and the nutritional risk, measured by the DNH score. A study that assessed EatC in a geographically diverse sample of individuals with metabolic syndrome, conducted in the United States among adults with a mean age of 55.5 years, shows that competent eaters consume significantly more vegetables and fruits, and that incorporating these foods into their daily routine is a more automatic and natural habit for them [51]. Brazilian and Finnish studies show that higher or improving EatC was associated with greater fruit and vegetable consumption and more regular meal frequency [32,52]. Furthermore, the literature confirms that individuals with greater EatC and positive eating behaviors tend to consume a higher-quality diet, with greater food diversity and nutritional balance [45]. This variety in the diet, promoted by EatC, may act as an important protective factor in old age, which may explain the negative association found between salad or raw vegetable consumption and the nutritional risk.

### 4.4. Associations Between Changes After the 12-Month Intervention

The food acceptance domain showed a negative correlation with changes in DNH, indicating an association between increased food acceptance and reduced nutritional risk. This is reinforced by a Brazilian study that examined the relationships among cooking and food skills and EatC in adults, suggesting that the ability to overcome the fear of trying new foods is essential for incorporating a more varied range of ingredients into the diet, potentially helping to promote a diverse diet [25]. Furthermore, these findings are supported by another Brazilian study that examined the associations among EatC, food consumption, and health outcomes. Individuals with higher EatC demonstrated greater confidence in their food choices, greater openness to trying new foods, and a greater ability to maintain a healthy balance between food preferences, food choices, and food intake [32].

Conversely, changes in internal regulation scores were positively correlated with DNH, indicating that participants who increased their internal regulation also had increased nutritional risk. This finding highlights a significant challenge in managing older adults with T2DM. A possible explanation is that when patients try to eat guided exclusively by hunger and satiety signals, without a previously established eating structure, this may be associated with poor food choices and skipping meals. A Brazilian study assessing the impact of T2DM on older adults’ quality of life shows that most already struggle to adhere to dietary restrictions, have lost motivation, and report not worrying about what they choose to eat [8]. This information is reinforced by a study that examines the relationship between cooking and food skills and EatC, suggesting that if internal regulation is not accompanied by the development of contextual skills (which allow individuals to plan menus, shopping, and the food environment), the recognition of hunger and satiety cues alone may not be sufficient [25]. Therefore, future interventions targeting older adults with type 2 diabetes or prediabetes should prioritize strengthening contextual skills, such as meal planning and the establishment of structured eating routines.

Furthermore, changes in soda or artificial juice consumption correlated positively with changes in nutritional risk, indicating that participants who increased their consumption of these beverages also showed increased nutritional risk. High consumption of sugar-sweetened beverages is directly linked to worsening of existing cases and the emergence of new cases of T2DM and cardiovascular disease, as demonstrated by a global epidemiological study that evaluated dietary data across 184 countries [53]. This scenario may explain the positive association between the increased consumption of sodas and artificial juices and the nutritional risk identified in the present study, consistent with current epidemiological evidence [53].

As stated in the Brazilian Diabetes Society recommendations on nutritional therapy for prediabetes and T2DM, these beverages should be avoided because they are associated with a higher risk of developing or worsening the disease [17].

### 4.5. Associations with Clinical Outcomes and Their Variations After the 12-Month Intervention

The analysis of EatC scores and clinical outcomes showed that internal regulation was positively correlated with BMI at the first collection. This result contrasts with most of the literature, which reports an inverse association. For example, a Finnish study of adults at increased risk of type 2 diabetes found that individuals with higher EatC had significantly lower BMI and waist circumference, reinforcing the consistent association between greater EatC and more favorable anthropometric outcomes [54]. A possible explanation for this baseline finding is that, in older adults with type 2 diabetes or prediabetes, long-term overweight may alter the physiological mechanisms that regulate hunger and satiety, making these internal cues more difficult to perceive. In addition, individuals with higher BMI may be more likely to follow strict dietary rules because of their diabetes and, during the initial assessment, may have mistaken this dietary control for true internal regulation.

Variation in contextual skills scores is negatively correlated with the variation in body fat percentage. This indicates that individuals who increased their contextual skills also showed a decrease in their body fat percentage, corroborating a review of health and non-weight-centric eating behaviors reports that higher EatC scores are directly associated with lower adiposity measures, including reduced body fat percentage [45]. In practice, competence in contextual skills may provide the organizational structure essential for mealtime routines, helping to prevent individuals from consuming excessive amounts of unhealthy foods. These foods are typically low in nutrients such as fiber, vitamins, and minerals, and high in calories, which may reduce satiety and promote excessive calorie intake and their high consumption is associated with the increase in the risk of develop obesity [18]. For this reason, structured programs focused on lifestyle changes that reinforce the importance of meal planning and organization are recommended to improve treatment adherence and body composition in people with T2DM or prediabetes [17]. Additional evidence reinforcing the importance of contextual skills was shown in a Finnish study that investigated changes in EatC during a 1-year intervention with adults at increased risk of type 2 diabetes. The study found that contextual skills were among the domains most strongly associated with better diet quality, lower body mass index and smaller waist circumference [52]. These findings suggest that strengthening contextual skills may be an important target for future lifestyle interventions.

### 4.6. Strengths and Limitations

The longitudinal design enabled evaluation of temporal changes in EatC and associated outcomes within a primary health care setting. The assessment of the subjects, which integrated behavioral, nutritional, and clinical variables, provided a comprehensive view of participants’ progress. Using the ecSI2.0™BR, a validated instrument adapted for the Brazilian population, strengthens the rigor of EatC assessment. Additionally, because the study was conducted within the public health system, it increases its relevance to similar contexts and helps fill a knowledge gap in Brazil.

Regarding limitations, follow-up losses reduced the initial sample of 38 participants to 33, limiting the generalizability of the findings. In addition, the absence of a control group prevents causal inference, and the predominance of women (87.9%) limits generalizability to men. Furthermore, because dietary intake data were collected using the food consumption markers from the VIGITEL questionnaire, a self-reported instrument subject to recall bias among older adults, it may not fully capture actual dietary patterns. The high proportion of eating-competent subjects at baseline (84.8%) may also have limited increases in EatC scores. Lastly, because recruitment of the participants also depended on telephone contact, and those who did not answer the calls were excluded, we may have faced a selection bias. No detailed information was collected about glucose-lowering medications or changes in treatment during follow-up. The numerous correlation analyses conducted in this study should be considered exploratory. Given the number of associations examined, the possibility of chance findings cannot be excluded. Therefore, the observed correlations should be regarded as hypothesis-generating and require confirmation in future studies.

This study’s results can guide future research on EatC in older adults with T2DM or prediabetes. Findings on contextual skills and food acceptance can serve as a starting point for studies that develop interventions focused on cooking skills and organizing daily food-related activities. Studies with control groups, larger samples, and participants from different locations could help assess if these interventions help improve EatC and nutritional and clinical outcomes. Also, including participants with different baseline EatC scores and assessing them at different times over a longer follow-up period could help us understand how these changes happen and if they last over time in this population.

## 5. Conclusions

This is the first longitudinal study to evaluate EatC among older adults with T2DM or prediabetes participating in a structured food and nutrition education program in Brazilian public Primary Health Care. The central hypothesis was partially confirmed. Although the total EatC score did not significantly improve over the 12-month follow-up, changes in specific EatC domains, particularly contextual skills and food acceptance, were associated with more favorable nutritional and clinical outcomes among older adults with T2DM or prediabetes participating in a structured food and nutrition education program. In addition, body fat percentage decreased over the intervention period despite stable BMI and glycated hemoglobin. These findings suggest that changes in specific dimensions of EatC may be more sensitive than the overall score for identifying meaningful behavioral changes and their relationships with nutritional outcomes. This study may contribute to the growing evidence supporting the potential role of EatC in diabetes and prediabetes care. Further controlled studies with larger samples are warranted to confirm these findings and clarify the causal pathways involved.

## 6. Statement on the Use of Artificial Intelligence

Generative Artificial Intelligence and AI-assisted tools (ChatGPT [GPT-6.1 Sol], Claude [Sonnet 5.5], Gemini [4 Argon], and Connected Papers [web version, accessed on 28 September 2026]) were used in the preparation of this text. The authors used ChatGPT, Claude, and Gemini to translate the content, originally written in Brazilian Portuguese, into American English. They also assessed coherence and cohesion and compared the numerical values presented in the results section in paragraph form with those shown in the tables to identify possible transcription or typing errors. The authors used Connected Papers, an AI-assisted tool, to explore the scientific literature on food literacy and map relevant publications. The authors independently evaluated and selected all references included in the manuscript and assume full responsibility for the final content, including any inaccuracies, as well as for the overall accuracy of the study.

## Figures and Tables

**Table 1:** Mean, standard deviation (SD) and *p*-value of eating competence (ecSI2.0™BR) and nutritional risk (DNH) scores according to age, education and occupation.

	Eating Competence					Nutritional Risk
	Eating Attitudes	Food Acceptance	Internal Reg.	Contextual Skills	TOTAL	DNH
Age *						
Up to 69 (*n* = 21)	13.81 (3.09)	7.10 (2.14)	4.95 (1.24)	11.48 (3.83)	37.33 (7.12)	9.14 (3.14)
70 or older (*n* = 11)	15.73 (2.10)	5.91 (2.34)	4.55 (1.29)	12.64 (1.80)	38.82 (4.00)	9.55 (4.68)
*p* **	0.076	0.160	0.393	0.352	0.529	0.773
Education *						
Up to 8 years (*n* = 16)	15.13 (2.42)	5.50 (2.37)	5.00 (1.32)	13.13 (1.96)	38.75 (5.03)	9.38 (3.83)
9 years or more (*n* = 16)	13.81 (3.27)	7.88 (1.36)	4.63 (1.20)	10.63 (3.90)	36.94 (7.23)	9.19 (3.62)
*p* **	0.207	0.002	0.407	0.032	0.417	0.888
Occupation *						
Inactive/retired (*n* = 24)	13.79 (2.84)	6.50 (2.32)	4.58 (1.32)	11.54 (3.27)	36.42 (6.09)	9.67 (4.01)
Active (*n* = 8)	16.50 (2.14)	7.25 (2.05)	5.50 (0.76)	12.88 (3.36)	42.13 (4.49)	8.13 (2.17)
*p* **	0.020	0.423	0.024	0.329	0.021	0.311

* One individual did not report this variable; ** Unpaired Student’s t test; Note: ecSI2.0™BR and DNH values collected at baseline.

**Table 2:** Temporal evolution of eating competence (ecSI2.0™BR), nutritional risk (DNH), and food consumption markers from baseline to 12 months (*n* = 33).

	0 Months	12 Months	*p*
BMI; mean (SD)	28.42 (5.26)	28.32 (5.88)	0.643 **
Body fat percentage *; mean (SD)	39.85 (7.21)	38.47 (8.46)	0.011 **
Glycated hemoglobin *; mean (SD)	6.09 (0.65)	6.09 (0.66)	0.971 **
Eating Competence (score)			
Eating Attitudes; mean (SD)	14.52 (2.87)	15.36 (2.79)	0.127 **
Food Acceptance; mean (SD)	6.64 (2.23)	6.30 (2.47)	0.534 **
Internal Reg.; mean (SD)	4.85 (1.25)	4.85 (1.27)	1.000 **
Contextual Skills; mean (SD)	11.94 (3.26)	12.27 (2.53)	0.555 **
TOTAL; mean (SD)	37.94 (6.12)	38.79 (4.88)	0.434 **
Eating Competence (categorized)			
ecSI2.0™BR ≥ 32; freq. (%)	28 (84.8%)	31 (93.9%)	0.375 ***
Nutritional risk (score)			
DNH; mean (SD)	9.27 (3.61)	8.48 (4.10)	0.221 **
Nutritional risk (categorized)			
(0–2) Good; freq. (%)	0 (0%)	1 (3.0%)	
(3–5) Moderate risk; freq. (%)	4 (12.1%)	7 (21.2%)	0.096 ****
(6 or more) High risk; freq. (%)	29 (87.9%)	25 (75.8%)	
Healthy eating markers			
Bean consumption			
Never/rarely; freq. (%)	3 (9.1%)	1 (3.0%)	
1 to 4 days per week; freq. (%)	6 (18.2%)	10 (30.3%)	1.000 ****
5 days or more per week; freq. (%)	24 (72.7%)	22 (66.7%)	
Vegetable consumption			
Never/rarely; freq. (%)	0 (0%)	1 (3.0%)	
1 to 4 days per week; freq. (%)	5 (15.2%)	2 (6.1%)	0.655 ****
5 days or more per week; freq. (%)	28 (84.8%)	30 (90.9%)	
Salad or raw vegetable consumption			
Never/rarely; freq. (%)	1 (3.0%)	0 (0%)	
1 to 4 days per week; freq. (%)	10 (30.3%)	10 (30.3%)	0.564 ****
5 days or more per week; freq. (%)	22 (66.7%)	23 (69.7%)	
Natural fruit juice consumption			
Never/rarely; freq. (%)	11 (33.3%)	14 (42.4%)	
1 to 4 days per week; freq. (%)	14 (42.4%)	12 (36.4%)	0.403 ****
5 days or more per week; freq. (%)	8 (24.2%)	7 (21.2%)	
Fruit consumption			
Never/rarely; freq. (%)	0 (0%)	1 (3.0%)	
1 to 4 days per week; freq. (%)	9 (27.3%)	6 (18.2%)	0.705 ****
5 days or more per week; freq. (%)	24 (72.7%)	26 (78.8%)	
Soda or artificial juice consumption			
Never/rarely; freq. (%)	26 (78.8%)	27 (81.8%)	
1 to 4 days per week; freq. (%)	7 (21.2%)	5 (15.2%)	1.000 ****
5 days or more per week; freq. (%)	0 (0%)	1 (3.0%)	

* One individual did not report this variable; ** Paired Student’s t test; *** McNemar test; **** Wilcoxon test.

**Table 3:** Pearson correlation coefficients and *p*-values between eating competence (ecSI2.0™BR) and nutritional risk (DNH) scores in the two collection periods (*n* = 33).

	Nutritional Risk (DNH)	
Eating Competence (ecSI2.0™BR)	0 Months Corr. (*p*)	12 Months Corr. (*p*)
Eating Attitudes	−0.192 (0.285)	−0.275 (0.122)
Food Acceptance	−0.224 (0.210)	−0.123 (0.496)
Internal Reg.	−0.094 (0.602)	−0.063 (0.728)
Contextual Skills	−0.155 (0.388)	−0.058 (0.747)
TOTAL	−0.274 (0.123)	−0.266 (0.135)

**Table 4:** Spearman correlation (and *p*-value) between healthy eating markers and total ecSI2.0™BR and DNH scores in each of the two collection periods (*n* = 33).

	Eating Competence (ecSI2.0™BR)		Nutritional Risk (DNH)	
Healthy Eating Markers (VIGITEL)	0 Months Corr. (*p*)	12 Months Corr. (*p*)	0 Months Corr. (*p*)	12 Months Corr. (*p*)
Beans	0.078 (0.667)	0.160 (0.375)	−0.154 (0.393)	−0.245 (0.170)
Vegetables	0.459 (0.007) *	0.091 (0.613)	−0.098 (0.586)	0.177 (0.323)
Salad or raw vegetable	0.420 (0.015) *	0.223 (0.213)	−0.161 (0.370)	−0.484 (0.004) *
Natural fruit juice	0.162 (0.368)	0.145 (0.420)	−0.060 (0.742)	−0.295 (0.096)
Fruits	0.304 (0.085)	0.286 (0.107)	−0.296 (0.094)	−0.201 (0.261)
Soda or artificial juice	−0.133 (0.461)	−0.029 (0.873)	0.085 (0.639)	0.103 (0.568)

* Statistically significant (p < 0.05).

**Table 5:** Pearson correlation (and *p*-value) between the evolution (variation between 0 and 12 months collection) of ecSI2.0™BR and DNH scores (*n* = 33).

	Nutritional Risk (Variation DNH **)
Eating Competence (Variation ecSI2.0™BR **)	Corr. (*p*)
Eating Attitudes	0.053 (0.771)
Food Acceptance	−0.417 (0.016) *
Internal Reg.	0.492 (0.004) *
Contextual Skills	0.047 (0.793)
TOTAL	−0.048 (0.793)

* Statistically significant (p < 0.05); ** Variation = 12-month value − 0-month value.

**Table 6:** Spearman correlation (and *p*-value) between the evolution of healthy eating markers and variations in ecSI2.0™BR and DNH scores (*n* = 33).

Healthy Eating Markers (Variation VIGITEL **)	Eating Competence (Variation ecSI2.0™BR **)	Nutritional Risk (Variation DNH **)
Beans	0.160 (0.375)	−0.284 (0.110)
Vegetables	0.107 (0.552)	0.026 (0.886)
Salad or raw vegetable	0.100 (0.580)	−0.039 (0.826)
Natural fruit juice	0.133 (0.462)	−0.023 (0.898)
Fruits	−0.003 (0.985)	0.065 (0.721)
Soda or artificial juice	0.152 (0.399)	0.503 (0.003) *

* Statistically significant (p < 0.05); ** Variation = 12-month value − 0-month value.

**Table 7:** Pearson correlation (and *p*-value) of ecSI2.0™BR and DNH scores with BMI, % body fat, and Glycated Hemoglobin (HbA1c) in each of the two collection periods.

		0 Months			12 Months	
	BMI (*n* = 33) Corr. (*p*)	% Body Fat (*n* = 32) Corr. (*p*)	HbA1c (*n* = 32) Corr. (*p*)	BMI (*n* = 33) Corr. (*p*)	% Body Fat (*n* = 32) Corr. (*p*)	HbA1c (*n* = 32) Corr. (*p*)
Eating Competence						
Eating Attitudes	0.125 (0.490)	−0.023 (0.900)	−0.024 (0.897)	−0.053 (0.770)	−0.140 (0.446)	0.102 (0.578)
Food Acceptance	−0.026 (0.885)	−0.178 (0.331)	0.005 (0.977)	0.057 (0.754)	−0.075 (0.684)	0.072 (0.694)
Internal Reg.	0.424 (0.014) *	0.120 (0.514)	−0.100 (0.586)	0.176 (0.328)	−0.025 (0.890)	−0.017 (0.925)
Contextual Skills	0.037 (0.838)	−0.213 (0.242)	0.267 (0.139)	−0.110 (0.541)	−0.204 (0.262)	−0.041 (0.822)
TOTAL	0.155 (0.388)	−0.164 (0.369)	0.112 (0.541)	−0.013 (0.944)	−0.228 (0.209)	0.069 (0.707)
Nutritional risk						
DNH	−0.009 (0.962)	0.317 (0.077)	0.055 (0.766)	0.057 (0.754)	0.299 (0.097)	0.201 (0.271)

* Statistically significant (p < 0.05).

**Table 8:** Pearson correlation (and *p*-value) between the evolution (variation between 0 and 12 months) of ecSI2.0™BR and DNH scores with the evolution of BMI, % body fat, and Glycated Hemoglobin (HbA1c).

	Variation in BMI ** (*n* = 33) Corr. (*p*)	Variation in % Body Fat ** (*n* = 32) Corr. (*p*)	Variation in HbA1c ** (*n* = 32) Corr. (*p*)
**Eating Competence** (Variation ecSI2.0™BR **)			
Eating Attitudes	−0.048 (0.790)	−0.067 (0.715)	−0.278 (0.123)
Food Acceptance	0.132 (0.463)	−0.026 (0.889)	0.178 (0.329)
Internal Reg.	0.127 (0.481)	0.100 (0.588)	−0.112 (0.541)
Contextual Skills	−0.084 (0.641)	−0.412 (0.019) *	−0.051 (0.783)
TOTAL	0.025 (0.889)	−0.239 (0.187)	−0.102 (0.579)
**Nutritional risk** (variation DNH **)			
DNH	0.163 (0.363)	0.263 (0.146)	0.001 (1.000)

* Statistically significant (*p* < 0.05); ** *Variation = 12-month value − 0-month value.*

## Data Availability

The data supporting the conclusions of this study are not public due to privacy and confidentiality restrictions regarding the participants as established in the informed consent form.

## References

[1] International Diabetes Federation (2025). IDF Diabetes Atlas 2025.

[2] Sociedade Brasileira de Diabetes (2025). Dados Epidemiológicos do Diabetes No Brasil 2024–2025.

[3] Sociedade Brasileira de Diabetes (2021). Diretrizes da Sociedade Brasileira de Diabetes 2019–2020.

[4] Magliano D., Boyko E. (2021). IDF Diabetes Atlas 10th edition scientific committee. International Diabetes Federation.

[5] Dos Reis R.C.P., Duncan B.B., Malta D.C., Iser B.P.M., Schmidt M.I. (2022). Evolution of Diabetes in Brazil: Prevalence Data from the 2013 and 2019 Brazilian National Health Survey.

[6] Brasil. Ministério da Saúde (2024). Vigitel Brasil 2023: Vigilância de Fatores de Risco e Proteção para Doenças Crônicas Por Inquérito Telefônico. http://www.gov.br/saude/pt-br/composicao/svsa.

[7] Brasil VIGITEL BRASIL 2023; Ministério da Saúde, 2023. http://www.saude.gov.br/svs.

[8] Leite E.D.S., Lubenow J.A.M., Moreira M.R.C., Martins M.M., da Costa I.P., Silva A.O. (2014). Avaliação do impacto da diabetes mellitus na qualidade de vida de idosos/Evaluation of the impact of diabetes mellitus on the quality of life of aged people. Ciência Cuid. E Saúde Univ. Estadual De Mar..

[9] dos Santos W.P., de Freitas F.B.D., Soares R.M., Souza G.d.A., Campos P.d.S., Bezerra C.d.O., Ramos K.S., Barbosa L.d.S. (2020). Complicações do diabetes mellitus na população idosa. Braz. J. Dev..

[10] Moura F., Salles J.E.N., Valente F., de Almeida-Pititto B., Fonseca R.M.C., Cavalcanti S., Bertoluci M. (2023). Abordagem Do Paciente Idoso Com Diabetes Mellitus.

[11] Rodrigues G.M.B., Malerbi F.E.K., Pecoli P.F.G., Forti A.E., Bertoluci M. (2022). Aspectos Psicossociais Do Diabetes Tipos 1 e 2.

[12] Costa P.d.A., de Oliveira Neta M.S., de Azevedo T.F., Cavalcanti L.T., Rocha S.d.S., Nogueira M.F. (2022). Emotional distress and adherence to self-care activities in older adults with diabetes mellitus. Rev. Rene Univ. Fed. Do Ceará (UFC).

[13] Rodacki M., Cobas R.A., Zajdenverg L., da Silva Júnior W.S., Giacaglia L., Calliari L.E., Noronha R.M., Valerio C., Custódio J., Scharf M. (2024). Diagnóstico de Diabetes Mellitus.

[14] Brasil. Ministério da Saúde (2024). Protocolo Clínico e Diretrizes Terapêuticas do Diabetes Mellitus Tipo 2. https://www.gov.br/conitec/pt-br.

[15] Harreiter J., Roden M. (2023). Diabetes mellitus: Definition, classification, diagnosis, screening and prevention (Update 2023). Wien. Klin. Wochenschr. Springer.

[16] Bahia L., de Almeida-Pititto B., Bertoluci M. (2024). Tratamento do DM2 no SUS.

[17] Ramos S., Campos L.F., Baptista D.R., Strufaldi M., Gomes D.L., Guimarães D.B., Souto D.L., Marques M., Sousa S.d.S., de Campos T.F. (2022). Terapia Nutricional no Pré-Diabetes e no Diabetes Mellitus Tipo 2. Dir. Of. Da Soc. Bras. De Diabetes.

[18] Ministério Da Saúde (2022). Orientação alimentar de pessoas adultas com diabetes mellitus. Protocolo de Uso do Guia Alimentar Para a População Brasileira.

[19] Ministério do Desenvolvimento Social e Combate à Fome (2012). Marco de Referência de Educação Alimentar e Nutricional Para as Políticas Públicas. https://cfn.org.br/wp-content/uploads/2017/03/marco_EAN.pdf.

[20] Brasil. Ministério da Saúde (2018). Princípios e Práticas para Educação Alimentar e Nutricional.

[21] Satter E. (2007). Eating Competence: Definition and Evidence for the Satter Eating Competence Model. J. Nutr. Educ. Behav..

[22] de Queiroz F.L.N., Nakano E.Y., Ginani V.C., Botelho R.B.A., Araújo W.M.C., Zandonadi R.P. (2020). Eating competence among a select sample of Brazilian adults: Translation and reproducibility analyses of the satter eating competence inventory. Nutr. MDPI AG.

[23] Kim J., Hur M.H. (2021). The effects of dietary education interventions on individuals with type 2 diabetes: A systematic review and meta-analysis. Int. J. Environ. Res. Public Health.

[24] De Queiroz F.L.N., Nakano E.Y., Botelho R.B.A., Ginani V.C., Cançado A.L.F., Zandonadi R.P. (2020). Eating competence associated with food consumption and health outcomes among brazilian adult population. Nutrients.

[25] Lins M., Nakano E.Y., Soares J.R.Q., de Queiroz F.L.N., Botelho R.B.A., Raposo A., Zandonadi R.P. (2024). Exploring the Relationship Between Cooking and Food Skills and Eating Competence Among Brazilian Adults. Nutrients.

[26] Boeira C.F., Nalon de Queiroz F.L., Zandonadi R.P., Rower H.B., Nakano E.Y., Feoli A.M.P. (2023). Eating Competence among Brazilian College Students. Int. J. Environ. Res. Public Health.

[27] de Queiroz F.L.N., Nakano E.Y., Botelho R.B.A., Ginani V.C., Raposo A., Zandonadi R.P. (2021). Eating competence among brazilian adults: A comparison between before and during the covid-19 pandemic. Foods.

[28] Brasil (2014). Guia Alimentar Para a População Brasileira Guia Alimentar Para a População Brasileira.

[29] Brasil (2018). Alimentação Cardioprotetora: Manual de Orientações Para Os Profissionais de Saúde Da Atenção Básica.

[30] Ministério da Saúde (2021). Fascículo 2 Protocolos de uso do Guia Alimentar Para a População Brasileira na Orientação Alimentar da População Idosa.

[31] Godleski S., Lohse B., Krall J.S. (2019). Satter Eating Competence Inventory Subscale Restructure After Confirmatory Factor Analysis. J. Nutr. Educ. Behav. Elsevier Inc..

[32] de Queiroz F.L.N., Raposo A., Han H., Nader M., Ariza-Montes A., Zandonadi R.P. (2022). Eating Competence, Food Consumption and Health Outcomes: An Overview. Int. J. Environ. Res. Public Health.

[33] Roediger M.D.A., Marucci M.D.F.N., Latorre M.D.R.D.D.O., Hearst N., De Oliveira C., Duarte Y.A.D.O., Lebrão M.L. (2017). Adaptação transcultural para o idioma português do método de triagem nutricional Determine your nutritional health^®^para idosos domiciliados. Ciênc. Saúde Coletiva.

[34] Nutrition Screening Initiative Determine Your Nutritional Health Checklist. https://umaine.edu/afuresources/wp-content/uploads/sites/679/2024/04/DETERMINE-Your-Nutritional-Health-Checklist.pdf.

[35] de Almeida Roediger M., de Fátima Nunes Marucci São Paulo M. Adaptação Transcultural e Avaliação das Propriedades Psicométricas do Determine Your Nutritional Health^®^, Para Idosos, No Municipio de São Paulo-SP, Brasil, 2015. https://teses.usp.br/teses/disponiveis/6/6138/tde-23112015-142051/publico/ManuelaDeAlmeidaRoediger.pdf.

[36] Texas Department of Aging and Disability Services Access & Intake/Area Agencies on Aging Instructions for Completing the Nutrition Risk Assessment (NRA) DETERMINE Your Nutritional Health Nutrition Screening Initiative (NSI) Background. https://www.hhs.texas.gov/sites/default/files/documents/doing-business-with-hhs/providers/health/nra.pdf.

[37] de Groot L.C.P.G.M., Beck A.M., Schroll M., van Staveren W.A. (1998). Evaluating the DETERMINE Your Nutritional Health Checklist and the Mini Nutritional Assessment as tools to identify nutritional problems in elderly Europeans. Eur. J. Clin. Nutr..

[38] Lipschitz D.A. (1994). Screening for Nutritional Status in the Elderly. Prim. Care Clin. Off. Pract..

[39] Gallagher D., Heymsfield S.B., Heo M., Jebb S.A., Murgatroyd P.R., Sakamoto Y. (2000). Healthy percentage body fat ranges: An approach for developing guidelines based on body mass index. Am. J. Clin. Nutr..

[40] Rush D., Mayer J. (1997). Nutrition Screening in Old People: Its Place in a Coherent Practice of Preventive Health Care. Annu. Rev. Nutr..

[41] World Health Organization (2011). Use of Glycated Haemoglobin (HbA1c) in the Diagnosis of Diabetes Mellitus: Abbreviated Report of a WHO Consultation.

[42] dos Reis J.C., de Mello D.N., Mascarenhas V., Giehl M.W.C. (2025). Self-reported diabetes and its complications in older adults in Brazil: Prevalence and correlates from the 2019 National Health Survey. Geriatr. Gerontol. Aging Soc. Bras. De Geriatr. E Gerontol..

[43] Flores T.R., Tomasi E., Neves R.G., de Oliveira Saes M., Silva Duro S.M. (2022). Inequalities in care for the people with diabetes in Brazil: A nationwide study, 2019. PLoS ONE.

[44] Maria da Silva J., Da Silva Fonseca J., Raquel Antunes Casimiro M., Barros de Quental O. (2025). Portadores de Diabetes: Controle, Adesão ao Tratamento e o Papel do sus na Atenção Primária. Rev. Interdiscip. Em Saúde Rev. Interdiscip. Em Saude.

[45] Eaton M., Probst Y., Foster T., Messore J., Robinson L. (2024). A systematic review of observational studies exploring the relationship between health and non-weight-centric eating behaviours. Appetite.

[46] Kipfer A., Partonen T., Laatikainen T., Mylläri J., Nuutinen O. (2025). Higher depressive symptoms are associated with lower eating competence among patients with moderate to severe depressive disorder. J. Psychiatr. Res..

[47] Bajaj M., McCoy R.G., Balapattabi K., Bannuru R.R., Bellini N.J., Bennett A.K., Beverly E.A., Briggs Early K., ChallaSivaKanaka S., Echouffo-Tcheugui J.B. (2026). 6. Glycemic Goals, Hypoglycemia, and Hyperglycemic Crises: Standards of Care in Diabetes—2026. Diabetes Care.

[48] Valente F., Lyra R., Albuquerque L., Cavalcanti S., Forti A.E., Bertoluci M. (2026). Manejo do Diabetes Mellitus Tipo 2. Diretriz da Sociedade Brasileira de Diabetes—Edição 2026. Conectando Pessoas. https://diretriz.diabetes.org.br/manejo-do-diabetes-mellitus-tipo-2/.

[49] Francisco P.M.S.B., de Assumpção D., Bacurau A.d.M., da Silva D.S.M., Yassuda M.S., Borim F.S.A. (2022). Diabetes mellitus in older adults, prevalence and incidence: Results of the FIBRA study. Rev. Bras. Geriatr. Gerontol..

[50] Moehlecke Iser B.P., Pinheiro P.C., Malta D.C., Duncan B.B., Schmidt M.I. (2021). Prediabetes and intermediate hyperglycemia prevalence in adults and associated factors, health national survey. Ciênc. Saúde Coletiva.

[51] Lohse B., Nguyen B., Drees B.M., Bailey-Davis L., Masters K.S., Nicklas J.M., Daniels B.T., Ruder E.H., Trabold N. (2024). Examination of Eating Competence in a Geo-diverse Sample with Metabolic Syndrome.

[52] Aittola K., Karhunen L., Männikkö R., Järvelä-Reijonen E., Mikkonen S., Absetz P., Kolehmainen M., Schwab U., Harjumaa M., Lindström J. (2021). Enhanced eating competence is associated with improved diet quality and cardiometabolic profile in finnish adults with increased risk of type 2 diabetes. Nutrients.

[53] Lara-Castor L., O’Hearn M., Cudhea F., Miller V., Shi P., Zhang J., Sharib J.R., Cash S.B., Barquera S., Micha R. (2025). Burdens of type 2 diabetes and cardiovascular disease attributable to sugar-sweetened beverages in 184 countries. Nat. Med. Nat. Res..

[54] Tilles-Tirkkonen T., Aittola K., Männikkö R., Absetz P., Kolehmainen M., Schwab U., Lindström J., Lakka T., Pihlajamäki J., Karhunen L. (2020). Eating Competence Is Associated with Lower Prevalence of Obesity and Better Insulin Sensitivity in Finnish Adults with Increased Risk for Type 2 Diabetes: The Stopdia Study. Nutrients.

